# Decentralized Consensus Protocols on *SO*(4)^*N*^ and *TSO*(4)^*N*^ with Reshaping

**DOI:** 10.3390/e27070743

**Published:** 2025-07-11

**Authors:** Eric A. Butcher, Vianella Spaeth

**Affiliations:** 1 Department of Aerospace and Mechanical Engineering, University of Arizona, 1130 N Mountain Avenue, Tucson, AZ 85721, USA; 2 Department of Mathematics, University of Arizona, 1130 N Mountain Avenue, Tucson, AZ 85721, USA; vspaeth@arizona.edu

**Keywords:** special orthogonal groups, consensus protocols, Morse–Lyapunov function, almost global asymptotic stability

## Abstract

Consensus protocols for a multi-agent networked system consist of strategies that align the states of all agents that share information according to a given network topology, despite challenges such as communication limitations, time-varying networks, and communication delays. The special orthogonal group SO(n) plays a key role in applications from rigid body attitude synchronization to machine learning on Lie groups, particularly in fields like physics-informed learning and geometric deep learning. In this paper, N-agent consensus protocols are proposed on the Lie group SO(4) and the corresponding tangent bundle TSO(4), in which the state spaces are $SO{\left(4\right)}^{N}$ and $TSO{\left(4\right)}^{N}$, respectively. In particular, when using communication topologies such as a ring graph for which the local stability of non-consensus equilibria is retained in the closed loop, a consensus protocol that leverages a reshaping strategy is proposed to destabilize non-consensus equilibria and produce consensus with almost global stability on $SO{\left(4\right)}^{N}$ or $TSO{\left(4\right)}^{N}$. Lyapunov-based stability guarantees are obtained, and simulations are conducted to illustrate the advantages of these proposed consensus protocols.

## 1. Introduction

In the past few decades, multi-agent systems have gained attention due to a wide range of applications, such as robotics, unmanned aerial vehicle swarms, and satellite formations. A key challenge is achieving consensus, where all agents in the network achieve a common (or desired relative) state, despite differences in initial conditions and communication constraints. Various approaches to the consensus problem have been pursued, such as consensus via optimization [1], where algorithms for global synchronization and balancing on connected compact manifolds become symmetric with respect to the absolute position on a manifold, allowing focus on the relative configuration. Geometric approaches for non-linear consensus problems are presented in [2], and global convergence issues not present in linear models are examined. Additional factors such as time delays in communication between agents may also be considered [3]. In all previous research on consensus, the importance of the communication topology is emphasized [4].

Lie groups, including SO(n), are used in machine learning methods by modeling transformations in a smooth and structured manner. Applications that require rotational invariance, such as robotics, computer vision, and physics simulations, can benefit from consensus-based distributed learning on SO(3), as presented in [5]. Decentralized learning frameworks can be employed to train models that respect the symmetries of rotational invariance and equivariance, such as in [6,7]. Consensus problems with multiple agents can learn representations that are equivariant to rotations, ensuring consistent performance regardless of orientation. For example, a reinforcement learning neural network on a robotic arm control system should be equivariant such that, if the arm’s position is rotated, the control outputs should rotate accordingly. Further discussion of this topic is presented in [8]. Some applications on the Lie group SO(4) in machine learning include representations of higher-dimensional rotations in theoretical physics and geometric deep learning, where data are represented on a manifold with higher-dimensional symmetries. The literature on this topic is limited, providing the opportunity for potential research in developing neural network architectures that are invariant to rotations in four dimensions. However, ref. [9] explores the continuity of rotation representations in neural networks and discusses the general rotation on SO(n). The mentioned paper provides insights into the methodologies of representing higher-dimensional neural networks, which can be useful when incorporating SO(4) symmetries in machine learning models.

Rotation matrices, which are elements of the Lie group SO(3), have gained interest in the development of attitude control techniques due to their non-singular and unique representation of rigid body attitude that results in globally continuous control laws [10]. Rigid body attitude consensus (or attitude synchronization) on $SO{\left(3\right)}^{N}$ and the tangent bundle $TSO{\left(3\right)}^{N}$ focuses on the cooperative control of the orientations of agents that share information within a network, ensuring that the multi-agent system can function in unison. The concept of consensus control on $SO{\left(n\right)}^{N}$ or its tangent bundle $TSO{\left(n\right)}^{N}$ can be interpreted with an understanding of attitude consensus on $SO{\left(3\right)}^{N}$ and $TSO{\left(3\right)}^{N}$. A thorough explanation of attitude using rotation matrices on SO(3) is presented in [10], including the use of Rodrigues’s formula, which maps the principal angle and axis to the rotation matrix, and Lyapunov-based stability analysis [10].

Various approaches have been taken to achieve consensus on special orthogonal groups. This includes a method similar to ref. [1] that reduces a non-linear consensus problem to the optimization of linear functionals over the convex hull of the group of rotation matrices on SO(n) [11]. Another optimization approach uses a gradient algorithm based on the cost function of an output space of a system and is applied to synchronization where only partial state information is shared [12]. This is useful in scenarios such as satellite communication links in a constellation where bandwidth is limited between neighboring lines. Similar to ref. [2], a geometric approach for coordinated motion in Lie groups is taken in [13], driving a swarm of simple integrator agents towards local convergence. In ref. [14], an analytical solution to closed-loop kinematics is presented and results in almost globally exponentially stabilizing control laws. An attitude tracking problem for a class of almost global asymptotic stability (AGAS) stable feedback laws is presented in [15] with a closed form solution as well, although non-consensus equilibria are not addressed. A leader–follower strategy for attitude formation tracking control is presented in [16] to achieve the desired relative attitude between agents, and the stability of the proposed tracking control law is enhanced by feedback reshaping. Similarly to the topologies explored in this paper, ref. [17] studies ring communication graphs and the almost global stabilization of consensus with input delays, destablizing undesired non-consensus equilibria using reshaping. Rigid body pose consensus control and the impact of communication delays between agents are studied in [18] on TSE(3), where rigid body attitudes are expressed as rotation matrices, and the almost global asymptotic stability of the consensus subspace is achieved by an extended Morse–Lyapunov–Krasovski approach. An extension of the Morse–Lyapunov approach is also used in [19] to achieve the almost global asymptotic stability of the consensus subspace on Lie groups SO(3) and SE(3).

In ref. [20], non-linear stability analysis and Lyapunov’s direct method are applied to design non-linear feedback control laws for rigid body control on TSO(3) and for the problem of multi-body attitude synchronization on $TSO{\left(3\right)}^{N}$. The improvement in globally continuous control laws using rotation matrices and the feedback reshaping strategy is demonstrated via stability analysis and simulation. Without reshaping, the attitude control of a rigid body with initial principal rotation angles (PRAs) close to 180° results in poor closed-loop response performance. Additionally, multi-body attitude synchronization on five- and nine-agent ring graphs results in stable non-consensus equilibria, whereas adding feedback reshaping destabilizes these equilibria and produces attitude synchronization with almost global stability. The authors find that applying a reshaping function for the PRA designed to destabilize the non-consensus equilibria ensures the almost global stability of the consensus subspace with a fixed number of agents. The authors observe that different numbers of agents may require different reshaping functions. Consensus with AGAS is achieved as long as the relative PRA of the undesired equilibrium is greater than the angle that maximizes the reshaping function. In this paper, four-dimensional rotations are used to represent an agent’s configuration, synonymous to an agent’s attitude in three dimensions, which is analagous to the application of rotation-invariant neural networks in four dimensions. Consensus protocols are proposed on the Lie group SO(4) and its corresponding tangent bundle TSO(4). These protocols are proven to achieve almost global asymptotic stability when applying the feedback reshaping strategy presented in [20].

The remainder of this paper is organized as follows. Section 2 provides the background on communication topologies and the properties of SO(4). Section 3 presents a proposed consensus protocol on $SO{\left(4\right)}^{N}$ with reshaping that destablizes non-consensus equilibria and achieves almost global asymptotic stability. Section 4 extends the consensus protocol to $TSO{\left(4\right)}^{N}$. Simulations on a five-agent ring graph and a nine-agent ring graph and ring lattice are subsequently presented. Finally, some conclusions are offered in Section 5.

## 2. Background

### 2.1. Communication Graph

Assume that there are *N* agents in a network represented by an undirected graph G=(V,E), where G is the graph, V={1,…,N} is the node set, and E⊆V×V is the edge set with $n_{e}$ edges. For agents i,j∈V, $A=[a_{ij}]$ is the N×N non-negative adjacency matrix of G, where $a_{ij}=1$ if agents *i* and *j* have a bidirectional communication path and $a_{ij}=0$ if not. In addition, it is assumed that there are no self-loops, so $a_{ii}=0$. The degree matrix of the graph is a diagonal matrix $D=diag(d_{ii})$ where

(1) $$d_{ii}=\sum_{k=1,k\neq i}^{N}a_{ik}.$$

The graph Laplacian is then defined as L=D−A. Given an arbitrary edge orientation of the undirected graph, an oriented incidence matrix B can be constructed of size $N\times n_{e}$, where $L=BB^{T}$. If the undirected graph is connected, then there is exactly one zero eigenvalue of the L matrix. The corresponding eigenvector, which is a vector of ones (possibly scaled by some real number), represents the consensus subspace. The proof involves recognition that the rows of L sum to zero. For an undirected and connected graph, the linear Laplacian flow $\dot{x}=-Lx$ solves the average consensus problem.

**Figure 1:**
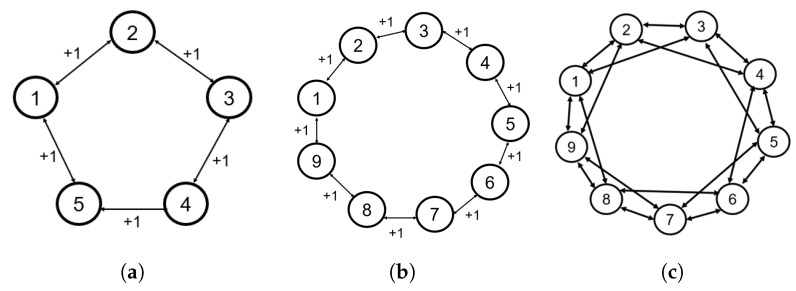
Three communication topologies: (**a**) 5-agent ring graph, (**b**) 9-agent ring graph, and (**c**) 9-agent ring lattice.

Ring graphs are a unique communication topology, known as difficult networks in which to achieve consensus or synchronization, as described in [21]. Ring graphs have equal numbers of edges to nodes, $n_{e}=N$. Ring lattices consist of nodes arranged in a ring, and each node is connected symmetrically to *m* of its nearest neighbors on each side. These graphs are illustrated in Figure 1.

The connectivity of a graph is defined as [21]

(2) $$\mu =\frac{\delta }{N-1}$$

where $\delta =\min(d_{ii})$ and N−1 is the maximum possible degree for any node in the *N*-agent graph. The connectivity ratio μ varies between 0 and 1, where a fully connected (complete) graph results in μ=1, an *N*-node ring graph has μ=2/(N−1), and an *N*-node ring lattice with m=2 has μ=4/(N−1). Specifically, the graphs in Figure 1 have connectivities of (a) μ=0.5, (b) μ=0.25, and (c) μ=0.5. The connectivity and symmetry properties of the network, along with the reshaping strategy to be introduced, influence the existence of any stable non-consensus equilibria in certain non-linear multi-agent systems on undirected graphs [21].

### 2.2. Properties of SO(4)

Rotations in higher-dimensional spaces have been a subject of considerable interest in fields such as mathematics, physics, and computer sciences. Methods of generating a rotation matrix in higher dimensions have been explored, including the use of Rodrigues’s formula and Cayley’s transforms, as discussed in [22,23,24,25]. This paper utilizes the Rodrigues formula to map the principal rotation angles (PRAs) and planes of rotation to an element on SO(4). The decomposition of a 4D rotation matrix in terms of an exponential skew-symmetric matrix is presented in [23,25,26] and is also leveraged here.

The intuitive three-dimensional rotation by a principal angle about a principal axis in SO(3) is replaced by planes of rotation in four dimensions on SO(4). Four-dimensional rotations can be decomposed into two PRAs and a set of two principal planes, which replace the principal angle and axis in SO(3). Methods to compute the PRAs and planes from a provided rotation matrix are discussed in [24], which is useful in classifying the rotation being applied. Meanwhile, ref. [25] presents an extensive discussion of the differences between simple, double, and isoclinic rotations. Algorithms to determine the PRAs, planes, and type of rotation are presented. This paper focuses on the most general case of double rotations to show how principal angles $\theta_{1,2}$, associated with the absolute or relative configurations, evolve under the consensus control problem.

Consider the 4×4 proper orthogonal matrix $R\in SO\left(4\right)=\{R^{4\times 4}|R^{T}R=I_{4},|R|=1\}$. Similarly to SO(3), this matrix represents a rotational transformation in four-dimensional Euclidean space $E^{4}$. The key difference between the dimensions is that, in three dimensions, the rotation is about an axis, whereas, in four dimensions, the rotation is about a plane. All vectors in the plane are mapped to other vectors in the same plane by the rotation and are related by corresponding eigenvalues and eigenvectors of the rotation matrix. Rotations in $E^{4}$ have at most two planes of rotations. The plane of rotation determines the classification of the rotation: (1) simple rotation—a single plane of rotation; (2) double rotation—two angles of rotation, each corresponding to one of two orthogonal planes of rotation. Isoclinic rotation is a specific type of double rotation where the non-zero angles of rotation about both planes are equal.

Let R∈SO(4) be a double rotation in the four-dimensional Euclidean space xyzw about the xy plane by angle $\theta_{2}$ and zw plane by angle $\theta_{1}$. Thus, the planes about which the rotations are performed are $E_{1}={\left[0_{2}\;I_{2}\right]}^{T}$ and $E_{2}={\left[I_{2}\;0_{2}\right]}^{T}$. This results in *R* in a particular basis with the normal form given by

(3) $$R=\left[\begin{array}{cccc} \cos\theta_{1} & -\sin\theta_{1} & 0 & 0\\ \sin\theta_{1} & \cos\theta_{1} & 0 & 0\\ 0 & 0 & \cos\theta_{2} & -\sin\theta_{2}\\ 0 & 0 & \sin\theta_{2} & \cos\theta_{2} \end{array}\right]$$

The convenience of the basis above is that the eigenvalues of the matrix are readily given by $\sigma \left(R\right)=\exp\left(\pm i\theta_{1}\right),\exp\left(\pm i\theta_{2}\right)$, where σ() is the spectrum operator and $\theta_{1}$ and $\theta_{2}$ are the PRAs. Equation (3) can be decomposed into two rotations $R_{1},R_{2}\in SO\left(4\right)$ as $R=R_{1}R_{2}=R_{2}R_{1}=R_{1}+R_{2}-I_{4}$, where $\sigma \left(R_{j}\right)=\left\{\exp\left(\pm i\theta_{j}\right),1,1\right\}$, j=1,2. The characteristic equation for a general R∈SO(4) can be easily obtained from Equation (3) as

(4) $$\det\left(R-\lambda I_{4}\right)=\lambda^{4}-2\left(\cos\theta_{1}+\cos\theta_{2}\right)\lambda^{3}+2\left(1+2\cos\theta_{1}\cos\theta_{2}\right)\lambda^{2}-2\left(\cos\theta_{1}+\cos\theta_{2}\right)\lambda +1=0$$

The comparison of Equation (4) with the standard form of the characteristic equation of a 4×4 proper orthogonal matrix given by

(5) $$\lambda^{4}-\mathrm{tr}\left(R\right)\lambda^{3}+{\mathrm{tr}}_{2}\left(R\right)\lambda^{2}-\mathrm{tr}\left(R\right)\lambda +1=0$$

results in the first- and second-order traces defined in terms of the PRAs as

(6) $$\mathrm{tr}\left(R\right)=\sum_{i=1}^{4}R_{ii}=2\left(\cos\theta_{1}+\cos\theta_{2}\right)\;$$

and

(7) $${\mathrm{tr}}_{2}\left(R\right)=\sum_{i=1}^{4}\sum_{j=i+1}^{4}|\begin{array}{cc} R_{ii} & R_{ij}\\ R_{ji} & R_{jj} \end{array}|=2\left(1+2\cos\theta_{1}\cos\theta_{2}\right).$$

The PRAs are then obtained by simultaneously solving Equations (6) and (7) as

(8) $$\cos\theta_{1,2}=\frac{\mathrm{tr}\left(R\right)\pm \sqrt{{\mathrm{tr}}^{2}\left(R\right)-4{\mathrm{tr}}_{2}\left(R\right)+8}}{4}$$

In the case of a simple rotation, $\theta =\theta_{1}$, $\theta_{2}=0$, cosθ=(tr(R)−2)/2. For an isoclinic rotation, $\theta_{1}=\theta_{2}\neq 0$, cosθ=tr(R)/4.

As presented in ref. [25], the Rodrigues formula can be extended from SO(3) to SO(4). The Rodrigues formula on SO(3) in terms of principal angle θ and principal axis $\hat{e}\in R^{3}$ with $∥\hat{e}∥=1$ is given by $R=\exp\left(\theta {\hat{e}}^{\times }\right)=I_{3}+\sin\theta {\left[\hat{e}\right]}^{\times }+\left(1-\cos\theta \right){\left({\left[\hat{e}\right]}^{\times }\right)}^{2}$, where ${\left(\right)}^{\times }$ is the cross-product operator that maps an element of $R^{3}$ to a 3×3 skew-symmetric matrix, ${\hat{e}}^{\times }\in \mathrm{so}\left(3\right)=\left\{S^{3\times 3}|S^{T}=-S\right\}$, and so(3) is the Lie algebra associated with the Lie group SO(3). Using this formulation as a guide, the Rodrigues formula on SO(4) can be obtained as follows. For a 4×4 skew-symmetric matrix $S\in \mathrm{so}\left(4\right)=\left\{S^{4\times 4}|S^{T}=-S\right\}$, where so(4) is the Lie algebra associated with the Lie group SO(4), and $\sigma \left(S\right)=\left(\pm i\theta_{1},\pm i\theta_{2}\right)$, the decomposition $S=\theta_{1}S_{1}+\theta_{2}S_{2}$, $S_{i}\in \mathrm{so}\left(4\right)$ holds such that $S_{i}^{3}=-S_{i}$, i=1,2, $S_{1}S_{2}=0$, $R_{i}=\exp\left(\theta_{i}S_{i}\right)$, and

(9) $$R=\exp\left(S\right)=\exp\left(\theta_{1}S_{1}+\theta_{2}S_{2}\right)=R_{1}R_{2}.$$

Using the properties of the matrix exponential, as well as the power series expansions of sin(x) and cos(x), this can be reduced to the Rodrigues formula on SO(4) given by

(10) $$R=I_{4}+\sin\theta_{1}S_{1}+\left(1-\cos\theta_{1}\right)S_{1}^{2}+\sin\theta_{2}S_{2}+\left(1-\cos\theta_{2}\right)S_{2}^{2}.\;$$

Using the formula $S_{i}^{2}=E_{i}E_{i}^{T}-I_{4}$, i=1,2, in terms of the planes of rotation $E_{1}$ and $E_{2}$, where $S_{i}E_{i}=0$, the symmetric component of *R* can be expressed as

(11) $$\frac{1}{2}\left(R+R^{T}\right)=\left(\cos\theta_{1}+\cos\theta_{2}-1\right)I_{4}+\left(1-\cos\theta_{1}\right)E_{1}E_{1}^{T}+\left(1-\cos\theta_{2}\right)E_{2}E_{2}^{T}\;$$

Equation (11) will be used in obtaining the classification of critical points of the Morse–Bott–Lyapunov function.

A standard form for matrix *S* in Equation (9) is [22]

(12) $$S=\left[\begin{array}{cc} a^{\times } & b\\ -b^{T} & 0 \end{array}\right]={\left[\begin{array}{c} a\\ b \end{array}\right]}^{\wedge }$$

where the ${\left(\right)}^{\vee }$ operator undoes the “wedge” ${\left(\right)}^{\wedge }$ operation, such that

(13) $$S^{\vee }=\left[\begin{array}{c} a\\ b \end{array}\right]\in R^{6}$$

The standard form for the characteristic equation of a 4×4 skew-symmetric matrix is

(14) $$\lambda^{4}+{\mathrm{tr}}_{2}\left(S\right)\lambda^{2}+\det\left(S\right)=0.\;$$

Using this and Equation (12), it can be shown that ${\mathrm{tr}}_{2}\left(S\right)=a^{T}a+b^{T}b$ and $\det\left(S\right)={\left(a^{T}b\right)}^{2}$ [22]. Using the basis assumed in Equation (3), the matrix *S* takes the real normal form given by

(15) $$S=\theta_{1}\left[\begin{array}{cc} J_{2} & 0_{2}\\ 0_{2} & 0_{2} \end{array}\right]+\theta_{2}\left[\begin{array}{cc} 0_{2} & 0_{2}\\ 0_{2} & J_{2} \end{array}\right],\;J_{2}=\left[\begin{array}{cc} 0 & -1\\ 1 & 0 \end{array}\right].$$

The characteristic equation for matrix *S* in Equation (15) is $\det\left(S-\lambda I_{4}\right)=\lambda^{4}+\left(\theta_{1}^{2}+\theta_{2}^{2}\right)\lambda^{2}+\theta_{1}^{2}\theta_{2}^{2}=0$. Comparing with Equation (14), the two PRAs are solved for as

(16) $$\theta_{1,2}=\sqrt{\frac{{\mathrm{tr}}_{2}\left(S\right)\pm \sqrt{{\mathrm{tr}}_{2}^{2}\left(S\right)-4\det\left(S\right)}}{2}}=\sqrt{\frac{a^{T}a+b^{T}b\pm \sqrt{{\left(a^{T}a+b^{T}b\right)}^{2}-4{\left(a^{T}b\right)}^{2}}}{2}}\;$$

In the case of a simple rotation where $\theta =\theta_{1}$, $\theta_{2}=0$, we have $\theta =\sqrt{{\mathrm{tr}}_{2}\left(S\right)}=\sqrt{a^{T}a+b^{T}b}$. For an isoclinic rotation where $\theta_{1}=\theta_{2}=\theta \neq 0$, we have $\theta =\sqrt{{\mathrm{tr}}_{2}\left(S\right)/2}=\sqrt{a^{T}b}$.

The challenge of representing the angular rate in four dimensions is discussed in [27]. The ability to represent the angular rate as a vector is unique to three dimensions. For four dimensions and above, the angular rate must be represented by a dyadic or tensor of the second rank. This becomes apparent when defining the four-dimension rotation matrix kinematics. The kinematic differential equation for R∈SO(4) is [14]

(17) $$\dot{R}=R\Omega \;$$

where

(18) $$\Omega ={\left[\begin{array}{c} \omega_{1}\\ \omega_{2} \end{array}\right]}^{\wedge }=\left[\begin{array}{cc} \omega_{1}^{\times } & \omega_{2}\\ -\omega_{2}^{T} & 0 \end{array}\right]\in \mathrm{so}\left(4\right)$$

and $\omega_{1},\omega_{2}\in R^{3}$. Note that, in ref. [14], the kinematics are presented as right-invariant, or $\dot{R}=\Omega R$, whereas this paper will maintain left-invariant kinematics. The dyadic Ω is a representation of the angular rate in four dimensions. Thus, Equation (17) corresponds to the kinematics on SO(3), namely $\dot{R}=R\omega^{\times }$, although ${\left(\omega_{1}^{T},\omega_{2}^{T}\right)}^{T}$ is not a physical angular velocity vector since the vector representation of the angular rate is unique to three dimensions, as emphasized in [27].

### 2.3. Morse–Bott–Lyapunov Function and Reshaping

In this section, the Morse–Bott–Lyapunov function on SO(4), along with its accompanying regulation protocol that drives an element of SO(4) to the identity matrix, is shown, and the reshaping strategy is introduced. These topics are preliminary to introducing the consensus protocols on $SO{\left(4\right)}^{N}$ and $TSO{\left(4\right)}^{N}$ in the following section. The derivation of the regulation protocol uses a Lyapunov-based approach in which the Lyapunov function, which is also a Morse–Bott function on SO(4), and its derivative have assumed forms in order to extract a regulation protocol.

Consider the Lyapunov candidate function, which is also a Morse–Bott function on SO(4), as will be shown, as

(19) $$V\left(R\right)=\frac{1}{2}\sum_{n=1}^{q}\frac{b_{n}}{n}\mathrm{tr}\left(I_{4}-R^{n}\right)>0\;\forall R\in SO\left(4\right)\setminus I_{4}\;$$

where *q* is the desired number of harmonics in the feedback protocol, and $b_{n}>0$ are positive coefficients that satisfy the following assumptions:

**Assumption 1.**  *$\sum_{n=1}^{q}nb_{n}\geq 1$;*

**Assumption 2.**  *$\sum_{n=1}^{q}{\left(-1\right)}^{n-1}nb_{n}=1$;*

**Assumption 3.**  *The only solutions of $f\left(\theta \right)=\sum_{n=1}^{q}b_{n}\sin\left(n\theta \right)=0$ are θ={0,π}mod2π.*

The simplest form of Equation (19) and the resulting protocol corresponds to $q=b_{1}=1$ and is called the Base case. The rationale behind using q>1 and selecting the $b_{n}$ coefficients is provided by the reshaping strategy, which will be explained subsequently.

Using the kinematics defined in Equation (17), the Lyapunov rate becomes

(20) $$\dot{V}\left(R\right)=-\frac{1}{2}\sum_{n=1}^{q}b_{n}\mathrm{tr}\left(R^{n}\Omega \right)={\left(\Omega^{\vee }\right)}^{T}\psi \left(R\right)\;$$

where

(21) $$\psi \left(R\right)=\sum_{n=1}^{q}b_{n}h\left(R^{n}\right).$$

and

(22) $$h\left(R\right)=\frac{1}{2}{\left(R-R^{T}\right)}^{\vee }=\sin\left(\theta_{1}\right)S_{1}^{\vee }+\sin\left(\theta_{2}\right)S_{2}^{\vee }$$

Setting the Lyapunov rate in Equation (20) equal to

(23) $$\dot{V}\left(R\right)=-{∥\psi \left(R\right)∥}^{2}\leq 0\;\forall R\in SO\left(4\right),\;$$

it is seen that Equation (19) is a Lyapunov function. Equations (20) and (23) result in the protocol

(24) $$\Omega =-\psi^{\wedge }\left(R\right)=-\sum_{n=1}^{q}b_{n}\left(\sin\left(n\theta_{1}\right)S_{1}+\sin\left(n\theta_{2}\right)S_{2}\right)$$

and the closed loop

(25) $$\dot{R}=-\frac{1}{2}\sum_{n=1}^{q}b_{n}\left(R^{n+1}-{\left(R^{T}\right)}^{n-1}\right)$$

Since Equation (19) has been established as a Lyapunov function, the conclusion can be made that $R=I_{4}$ is locally stable in Equation (25). Note that, in the Base case $V\left(R\right)=\mathrm{tr}\left(I_{4}-R\right)/2$, the protocol is $\Omega =-h^{\wedge }\left(R\right)$ and the closed loop is $\dot{R}=\left(I_{4}-R^{2}\right)/2$. The following theorem demonstrates that $R=I_{4}$ is almost globally asymptotically stable and that V(R) is also a Morse–Bott function.

**Theorem 1.**  *The Lyapunov function in Equation (19) is also a Morse–Bott function, while $R=I_{4}$ is almost globally asymptotically stable in Equation (25).*

**Proof.**  The variation in *R* with time held fixed is obtained from Equation (17) as

(26) δR=RΣ

where Σ∈so(4) is a variational matrix. Taking the variation of Equation (19) and using Equation (26) results in

(27) $$\delta V\left(R\right)=-\frac{1}{2}\sum_{n=1}^{q}b_{n}\mathrm{tr}\left(R^{n}\Sigma \right)={\left(\Sigma^{\vee }\right)}^{T}\psi \left(R\right)\;$$

The critical points of Equation (19) and the equilibria of Equation (25) can be analyzed by assessing δV(R)=0. Since Σ is an arbitrary variation, this is equivalent to ψ(R)=0, which results in $R=I_{4}$, and the submanifold of π rotations about any one or two planes $E_{1}$ and $E_{2}$, which is equivalent to the pair of PRAs $\left(\theta_{1},\theta_{2}\right)\in \left\{\left(0,\pi \right),\left(\pi ,0\right),\left(\pi ,\pi \right)\right\}$. To classify the critical points and determine the stability of these equilibria, the second variation of Equation (19) is obtained as

(28) $$\delta^{2}V\left(R\right)=-\frac{1}{2}\sum_{n=1}^{q}nb_{n}\mathrm{tr}\left(R^{n}\Sigma^{2}\right)=\frac{1}{2}{\left(\Sigma^{\vee }\right)}^{T}\Psi \left(R\right)\left(\Sigma^{\vee }\right)\;$$

where the Hessian is defined as

(29) $$\Psi \left(R\right)=\sum_{n=1}^{q}nb_{n}H\left(R^{n}\right)\;$$

and

(30) $$H\left(R\right)=\left[\begin{array}{cc} \mathrm{tr}\left(R_{11}\right)I_{3}-\frac{1}{2}\left(R_{11}+R_{11}^{T}\right) & \frac{1}{2}{\left(R_{12}+R_{21}^{T}\right)}^{\times }\\ -\frac{1}{2}{\left(R_{12}+R_{21}^{T}\right)}^{\times } & \frac{1}{2}\left(R_{11}+R_{11}^{T}\right)+R_{22}I_{3} \end{array}\right].$$

The matrices $R_{ij}$ are submatrices of *R* that result from partitioning as

(31) $$R=\left[\begin{array}{cc} R_{11} & R_{12}\\ R_{21} & R_{22} \end{array}\right]\;$$

and $R_{11}\in R^{3\times 3},R_{12}\in R^{3\times 1},R_{21}\in R^{1\times 3},R_{22}\in R$. Evaluating Ψ(R) at the critical points, the identity configuration is a Morse critical point with $\Psi \left(I_{4}\right)=2I_{6}\sum_{n=1}^{q}nb_{n}>0$. Similarly, the submanifolds of π rotations in $\theta_{1}$ and/or $\theta_{2}$ are non-Morse critical points, where using the normal form in Equation (3) and Assumptions 1 and 2 yields Ψ(diag([1,1,−1,−1])) as a diagonal matrix with matrix elements $\Psi_{\left(3,3\right)}>2$ and $\Psi_{\left(6,6\right)}=-2$, while Ψ(diag([−1,−1,1,1])) has $\Psi_{\left(3,3\right)}=-2$ and $\Psi_{\left(6,6\right)}>2$, both resulting in indefinite Ψ(R), while $\Psi (diag\left(\left[-1,-1,-1,-1\right]\right)=-2I_{6}$. Equation (11) can be used to calculate Ψ in terms of the planes of rotation $E_{1},E_{2}$. Since using different planes of rotation $E_{1}$ and $E_{2}$ would not change the eigenvalues of Ψ(R), it is seen that Ψ(R) is non-degenerate in the directions normal to the equilibrium manifold and degenerate along the tangent directions to the critical manifold, corresponding to rotations by π. Thus, Equation (33) is a Morse–Bott–Lyapunov function. Therefore, there is a neighborhood of $R=I_{4}$ where $\dot{V}\left(R\right)<0$, while, because the submanifolds of rotations by π constitute a set of zero measure in SO(4), the stability of $R=I_{4}$ in Equation (25) can be strengthened to almost global asymptotic stability (AGAS). □

Reshaping is a protocol strategy that has been presented in [16,20]. The coefficients *q* and $b_{n}$ are chosen as defined in [20], with the restrictions in Assumptions 1–3 for the Base case and two reshaping cases as follows:

- Base case (no reshaping): $q=b_{1}=1$;
- Case 1 reshaping: q=3, $\left(b_{1},b_{2},b_{3}\right)=\left(1.1819,0.4326,0.2278\right)$;
- Case 2 reshaping: q=5, $\left(b_{1},b_{2},b_{3},b_{4},b_{5}\right)=\left(1.1081,0.3872,0.2886,0.1676,0.0942\right)$.

**Figure 2:**
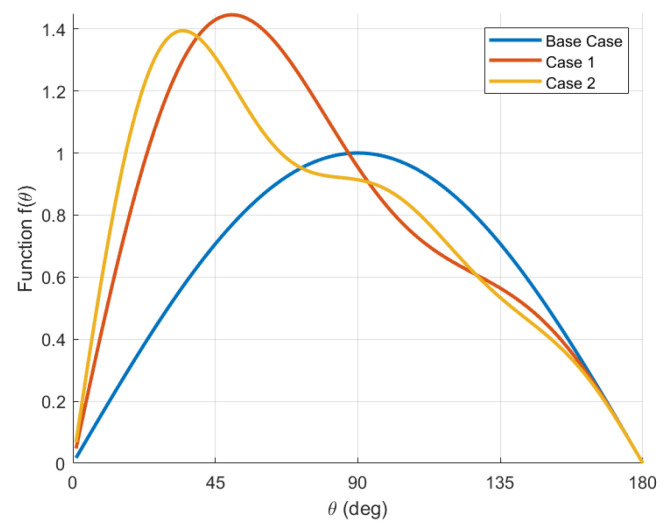
Function f(θ) for the Base case and reshaping cases 1 and 2.

Consider the function $f\left(\theta \right)=\sum_{n=1}^{q}b_{n}\sin\left(n\theta \right)$ in the reshaping strategy. This function is derived from the Fourier series expansion of an odd function of one of the principal rotation angles [20]. This function for the Base case and reshaping cases 1 and 2 is shown in Figure 2. Both presented reshaping functions defined by Fourier expansions show that the PRA associated with the maximum of f(θ) is decreased with respect to 90° for the Base case. Specifically, argmaxf(θ)=50° for Case 1 reshaping and 35° for Case 2 reshaping. Although the reshaping strategy does not affect the stability in Equation (25) for a single agent, it will be seen that the location of the maximum of f(θ) determines the stability of certain non-consensus equilibria of the closed-loop dynamics for the multi-agent problem. This is used to increase the region of attraction of the consensus manifold of the multi-agent system with the possibility of AGAS stability if no other equilibria are locally stable, as will be seen in the next section. As discussed earlier, the connectivity and symmetry of the network, along with the reshaping strategy, influence the existence of stable non-consensus equilibria in the closed loop for the multi-agent problem.

## 3. Consensus on *SO*(4)*^N^* with Reshaping

### 3.1. Proposed Consensus Protocol on $SO{\left(4\right)}^{N}$

Suppose that there are *N* agents that share information according to an undirected and connected communication graph. Let $R_{i}\in SO\left(4\right)$ be the configuration of agent *i* whose angular velocity tensor is $\Omega_{i}$. The kinematics associated with the *N* agents are

(32) $${\dot{R}}_{i}=R_{i}\Omega_{i}\;$$

for i=1,…,N. It is desired to drive the configurations to consensus asymptotically such that $R_{j}^{T}R_{i}=I_{4}$ for all (i,j) pairs in the steady state. As in the previous section, a Lyapunov-based approach is applied to obtain the consensus protocol by specifying the Lyapunov function (which is also a Morse–Bott function) and its derivative. For this purpose, consider the Morse–Bott–Lyapunov candidate function on $SO{\left(4\right)}^{N}$ as

(33) $$V\left(R_{j}^{T}R_{i}\right)=\frac{1}{2}\sum_{1\leq i<j\leq N}a_{ij}\sum_{n=1}^{q}\frac{b_{n}}{n}\mathrm{tr}\left(I_{4}-{\left(R_{j}^{T}R_{i}\right)}^{n}\right)>0\;\forall R_{j}^{T}R_{i}\in SO\left(4\right)\setminus I_{4}\;$$

where $a_{ij}$ are the elements of the adjacency matrix of the communication graph, and $R_{j}^{T}R_{i}$ is the relative configuration between agents *i* and *j*, for which the two principal rotation angles are $\theta_{ij1}$ and $\theta_{ij2}$. Using Equations (21) and (32), and the undirected property of the graph, the derivative of Equation (33) becomes

(34) $$\dot{V}\left(R_{j}^{T}R_{i}\right)=\sum_{i=1}^{N}{\left(\Omega_{i}^{\vee }\right)}^{T}\sum_{j=1}^{N}a_{ij}\psi \left(R_{j}^{T}R_{i}\right)\;$$

where

(35) $$\psi \left(R_{j}^{T}R_{i}\right)=\sum_{n=1}^{q}b_{n}\left(\sin\left(n\theta_{ij1}\right)S_{ij1}^{\vee }+\sin\left(n\theta_{ij2}\right)S_{ij2}^{\vee }\right).$$

Due to the form of Equation (34), it is convenient to choose

(36) $$\dot{V}\left(R_{j}^{T}R_{i}\right)=-k\sum_{i=1}^{N}∥\sum_{j=1}^{N}a_{ij}\psi \left(R_{j}^{T}R_{i}\right){∥}^{2}\leq 0\;\forall R_{j}^{T}R_{i}\in SO\left(4\right)\;$$

where k>0 is a positive gain, thus making Equation (33) a Lyapunov function. The resulting consensus protocol can be solved by equating Equations (34) and (36) as

(37) $$\Omega_{i}=-k\sum_{j=1}^{N}a_{ij}\psi^{\wedge }\left(R_{j}^{T}R_{i}\right)=-k\sum_{j=1}^{N}a_{ij}\sum_{n=1}^{q}b_{n}\left(\sin\left(n\theta_{ij1}\right)S_{ij1}\right)+\left(\sin\left(n\theta_{ij2}\right)S_{ij2}\right)$$

thus yielding the closed loop

(38) $${\dot{R}}_{i}=-\frac{k}{2}\sum_{j=1}^{N}a_{ij}\sum_{n=1}^{q}b_{n}R_{i}\left[{\left(R_{j}^{T}R_{i}\right)}^{n}-{\left(R_{i}^{T}R_{j}\right)}^{n}\right)],\;i=1,\ldots ,N.$$

Note that the consensus protocol in Equation (37) utilizes the relative Laplacian flow since it uses feedback of the relative configurations. This type of protocol is commonly used with undirected graphs and is required to achieve consensus with almost global stability on a compact manifold such as SO(4). A protocol that utilizes an absolute Laplacian flow [28] would be needed for the case of a directed graph, as was conducted in [29] for SO(3), although only local consensus stability would be achieved.

It will be shown in the next section that there are three possible classifications of equilibria. The consensus equilibrium where configuration synchronization is achieved results from the solution $R_{j}^{T}R_{i}=I_{4}$ for all (i,j) pairs, which implies that the relative configuration PRAs are $\theta_{ij1},\theta_{ij2}=0$ and Equation (33) achieves its minimum. The case of $\theta_{ijk}=\pi$ for at least one pair (i,j) for k=1 and/or k=2 while the remaining PRAs are $\theta_{ijk}=0$ yields an unstable set, resulting in partial consensus. In both of these cases, $\psi (R_{j}^{T}R_{i})=0$ for all (i,j) pairs. The third case results when at least one $\theta_{ijk}\neq 0$ or π, and this results when $\psi (R_{j}^{T}R_{i})\neq 0$ for at least one (i,j) pair, while the sum over *j* vanishes for i=1,…,N. As will be demonstrated in the simulation, the presence of stable non-consensus equilibria is dependent on the communication topology and reshaping case used. If stable non-consensus equilibria exist, the consensus configuration in Equation (38) is not AGAS stable but locally stable.

### 3.2. Local Stability Analysis

Consider the Rodrigues formula for the relative configuration

(39) $${\left(R_{j}^{T}R_{i}\right)}^{n}=\exp\left(nS_{ij}\right)=I_{4}+\sin\left(n\theta_{ij1}\right)S_{ij1}+\left(1-\cos\left(n\theta_{ij1}\right)\right)S_{ij1}^{2}+\sin\left(n\theta_{ij2}\right)S_{ij2}+\left(1-\cos\left(n\theta_{ij2}\right)\right)S_{ij2}^{2},$$

where $S_{ij}$ is the skew-symmetric matrix corresponding to relative configuration $R_{j}^{T}R_{i}$, which decomposes into $S_{ij}=\theta_{ij1}S_{ij1}+\theta_{ij2}S_{ij2}$, while $S_{ijk}^{2}=E_{ijk}E_{ijk}^{T}-I_{4}$, k=1,2. The variation of Equation (39) is

(40) $$\delta \left({\left(R_{j}^{T}R_{i}\right)}^{n}\right)=n{\left(R_{j}^{T}R_{i}\right)}^{n}\Sigma_{ij}=n{\left(R_{j}^{T}R_{i}\right)}^{n}\left(\Sigma_{i}-\Sigma_{j}\right)\;$$

where the last equality results from differencing infinitesimal variations. Using Equation (40), the first variation of the Lyapunov function in Equation (33) becomes

(41) $$\begin{array}{cc} \delta V(R_{j}^{T}R_{i}) & =-\frac{1}{2}\sum_{1\leq i<j\leq N}a_{ij}\sum_{n=1}^{q}b_{n}\mathrm{tr}\left({\left(R_{j}^{T}R_{i}\right)}^{n}\left(\Sigma_{i}-\Sigma_{j}\right)\right)\\ \; & =\sum_{1\leq i<j\leq N}a_{ij}{\left(\Sigma_{i}^{\vee }-\Sigma_{j}^{\vee }\right)}^{T}\psi \left(R_{j}^{T}R_{i}\right) \end{array}$$

and the second variation yields

(42) $$\begin{array}{cc} \delta^{2}V\left(R_{j}^{T}R_{i}\right) & =-\frac{1}{2}\sum_{1\leq i<j\leq N}a_{ij}\sum_{n=1}^{q}nb_{n}\mathrm{tr}\left({\left(R_{j}^{T}R_{i}\right)}^{n}{\left(\Sigma_{i}-\Sigma_{j}\right)}^{2}\right)\\ \; & =\sum_{1\leq i<j\leq N}a_{ij}{\left(\Sigma_{i}^{\vee }-\Sigma_{j}^{\vee }\right)}^{T}\Psi \left(R_{j}^{T}R_{i}\right)\left(\Sigma_{i}^{\vee }-\Sigma_{j}^{\vee }\right) \end{array}$$

where the Hessian of the second variation is

(43) $$\Psi \left(R_{j}^{T}R_{i}\right)=\sum_{n=1}^{q}nb_{n}H\left({\left(R_{j}^{T}R_{i}\right)}^{n}\right).$$

Consider the Base case, where q=1 and $b_{n}=1$ in Equations (33) and (37). Then, consider the stack column matrix $\Sigma ={\left({\left(\Sigma_{1}^{\vee }\right)}^{T}\;{\left(\Sigma_{2}^{\vee }\right)}^{T}\;\ldots \;{\left(\Sigma_{n}^{\vee }\right)}^{T}\right)}^{T}$. Denote the configuration of the multi-agent system as $R=\{R_{1},R_{2},\ldots ,R_{n}\}$ and rewrite $\delta V=\sum_{i<j}a_{ij}{\left(\Sigma_{i}^{\vee }-\Sigma_{j}^{\vee }\right)}^{T}h\left(R_{j}^{T}R_{i}\right)$ as $\delta V=\Sigma^{T}\gamma \left(R\right)$, $\gamma_{i}\left(R\right)=\sum_{j=1}^{N}{\left(B^{*}W_{h}^{*}\left(R\right)\right)}_{ij}$ where $B^{*}=B⨂I_{6}$, and $W_{h}^{*}\left(R\right)=diag\left(\ldots h\left(R_{j}^{T}R_{i}\right)\ldots \right)$ is the $6n_{e}\times n_{e}$ augmented weight matrix. Thus, the critical points of Equation (33) and relative equilibria in the closed loop of Equation (38) are obtained from γ(R)=0, while the classification of these points is obtained by rewriting $\delta V^{2}\left(R_{j}^{T}R_{i}\right)=\Sigma^{T}\Gamma \left(R\right)\Sigma /2$, where $\Gamma \left(R\right)=B^{*}W_{H}^{*}\left(R\right)B^{*T}$, and $W_{H}^{*}\left(R\right)=diag\left(\ldots H\left(R_{j}^{T}R_{i}\right)\ldots \right)$ is the $6n_{e}\times 6n_{e}$ augmented weight matrix that determines the classification.

In addition to relative configurations satisfying $(\theta_{ij1}$, $\theta_{ij2})=\left(0,0\right)$, (π,0),(0,π), or (π,π), the third category of equilibria mentioned above may exist. The balanced configuration of an *N*-agent ring graph results in Morse critical points of $\left\{\theta_{1},\theta_{2}\right\}\in \{\left\{0,2p_{2}\pi /N\right\},\left\{2p_{1}\pi /N,0\right\},\left\{2p_{1}\pi /N,2p_{2}\pi /N\right\}\}$, where $p_{1},p_{2}\in \left[1,\left\lfloor \frac{N}{2}\right\rfloor \right]$ are integers corresponding to balanced configurations. In the case of a ring graph or ring lattice, for instance, balanced equilibria (which may be stable or unstable) and unbalanced equilibria (always unstable) corresponding to rotations about the same plane(s) also exist. As in the single-agent case, the critical point classification and the stability of $R_{j}^{T}R_{i}=I_{4}$ depend on the spectrum of $H\left(I_{4}\right)=2I_{6}≻0$; therefore, the consensus manifold is locally stable. The partial consensus manifold is unstable since $H(\exp\left(\pi S_{ijk}\right))$, k=1 or 2, is indefinite, while $H(\exp\left(\pi S_{ij1}+\pi S_{ij2}\right)$ is negative definite. In the case of the five-agent ring graph with a balanced $p_{k}=1$ equilibrium where k=1 or 2, $H(R_{1}^{T}R_{2})$ is positive definite with spectrum $\sigma \left(H\left(R_{1}^{T}R_{2}\right)\right)=\left(0.62,1.3,2.0\right)$. Equal weights $a_{ij}$ have been assumed; therefore, local stability can be concluded based on a single pair of connected agents. However, if the weights are not equal, an assessment of the spectrum of Γ(R) or $W_{\Psi }^{*}\left(R\right)$ is required for a stability conclusion with different relative configurations between the agents.

The extension to the reshaping case can now be applied by modifying the augmented weight matrices $W_{\psi }^{*}\left(R\right)=diag\left(\ldots \psi \left(R_{j}^{T}R_{i}\right)\ldots \right)$ and $W_{\Psi }^{*}\left(R\right)=diag\left(\ldots \Psi \left(R_{j}^{T}R_{i}\right)\ldots \right)$. The stability of the consensus equilibrium $R_{j}^{T}R_{i}=I_{4}$ depends on the critical point classification, which, in this case, is solely driven by the spectrum of $\Psi (I_{4})$. In the case of the five-agent ring graph in the $p_{k}=1$ balanced equilibrium with Case 1 reshaping, $\sigma \left(\Psi \left(R_{j}^{T}R_{i}\right)\right)=\left(-1.78,1.84,5.46\right)$, confirming that the non-consensus equilibria have been destabilized. For the nine-agent ring graph with Case 1 reshaping, a balanced $p_{k}=1$ equilibrium is locally stable with $\sigma \left(\Psi \left(R_{j}^{T}R_{i}\right)\right)=\left(1.43,3.44,5.46\right)$. Case 2 reshaping results in an indefinite Ψ with $\sigma \left(\Psi \left(R_{j}^{T}R_{i}\right)\right)=\left(-1.04,3.37,7.78\right)$, thus yielding unstable balanced equilibria. Similarly, for the nine-agent ring lattice case with m=2, the balanced $p_{k}=1$ equilibrium for the Base case is locally stable with $\sigma \left(H\left(R_{1}^{T}R_{2}\right)\right)=\left(1.53,1.77,2.0\right)$ and $\sigma \left(H\left(R_{1}^{T}R_{3}\right)\right)=\left(0.35,1.17,2.0\right)$. For a ring lattice with m=2, two pairs of agents must be assessed in order to determine stability via the spectrum of the Hessian for these pairs. Unlike the ring graph, the relative configurations for edges between consecutive nodes (e.g., agents 1 and 2) and edges between non-consecutive nodes (e.g., agents 1 and 3) will not be equal; hence, the corresponding Ψ matrices are not equal. Case 1 reshaping results in $\sigma \left(\Psi \left(R_{1}^{T}R_{2}\right)\right)=\left(1.43,3.44,5.46\right)$ and $\sigma \left(\Psi \left(R_{1}^{T}R_{3}\right)\right)=\left(-1.90,1.78,5.46\right)$, and thus Case 1 reshaping yields an unstable balanced equilibrium for a ring lattice with m=2. Since $b_{n}$ have been chosen such that $b_{n}>0$, the consensus manifold is locally stable. Furthermore, since it is an isolated critical point, it is locally asymptotically stable. Equation (33) is a Morse–Bott–Lyapunov function for the same reasons as in the case of a single agent. If no other locally stable equilibria exist, then the consensus manifold is AGAS stable. As explained previously, reshaping can be used to enlarge the region of attraction of the consensus manifold and possibly render it AGAS stable.

### 3.3. Illustrative Examples

In this section, the significance and justification of the use of feedback reshaping is demonstrated for multi-agent consensus on SO(4). An undirected ring graph is used, while the initial conditions are chosen in the region of attraction of the consensus manifold, as well as of non-consensus equilibria. The Base case, reshaping Case 1, and reshaping Case 2 are all compared.

#### 3.3.1. Initial Conditions near Consensus

**Figure 3:**
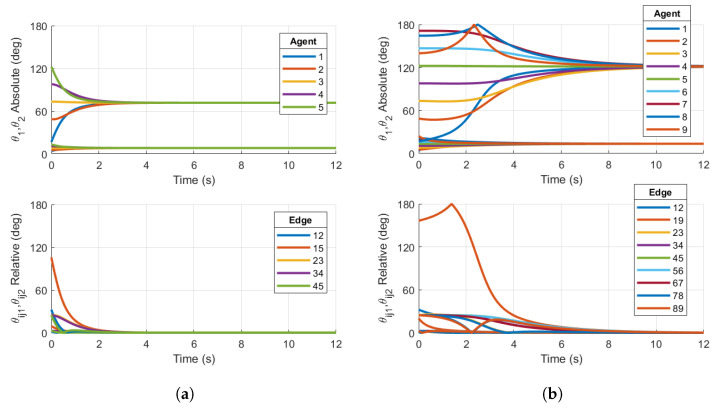
Simulations on $SO{\left(4\right)}^{N}$ for (**a**) Base case protocol on a 5-agent ring graph and (**b**) Base case protocol on a 9-agent ring graph. Initial conditions are chosen within the region of attraction of the consensus manifold. Both plots demonstrate consensus.

We first show simulations that demonstrate the local stability of the consensus manifold using the Base case protocol for the two ring graphs shown in Figure 1a,b. Figure 3 shows examples of the Base case for both five- and nine-agent ring graphs achieving consensus, where the initial conditions were specifically chosen within the region of attraction of the consensus manifold. The following simulations will demonstrate that the same topologies with certain initial conditions will result in convergence towards stable non-consensus equilibria.

#### 3.3.2. Five-Agent Ring Graph with Initial Conditions Away from Consensus

Now, we reconsider the case of the ring graph with N=5 agents in Figure 1a. The initial configurations of the agents are now assumed to be

(44) $$\begin{array}{c} R_{1}=expm\left(0.1S_{1}+0.2S_{2}\right)\;R_{i}=expm\left(\frac{\left(2i-2\right)}{N}\pi S_{1}+0.2iS_{2}\right),\;i=1,\ldots ,N\\ S_{1}={\left({\left[0\;0\;1\;0\;0\;0\right]}^{T}\right)}^{\wedge }\;S_{2}={\left({\left[0\;1\;0\;0\;0\;1\right]}^{T}\right)}^{\wedge } \end{array}$$

**Figure 4:**
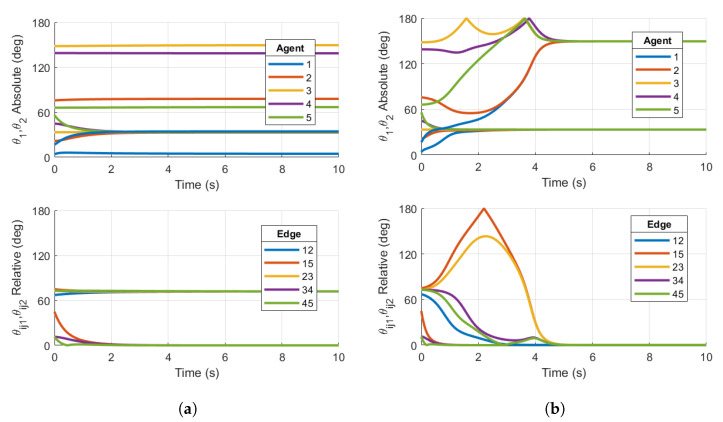
Simulations on $SO{\left(4\right)}^{5}$ for (**a**) Base case protocol on a 5-agent ring graph and (**b**) Case 1 reshaping protocol with $\arg\max_{\theta \in [0,180]}f\left(\theta \right)=50{}^{\circ}$ on a 5-agent ring graph. Initial conditions are chosen in the region of attraction of the p=1 non-consensus equilibria. Right plot demonstrates consensus, while left plot does not.

The skew-symmetric matrices have been selected to ensure that the conditions detailed in Section 2.2 are met, including the relation $S_{1}S_{2}=0$, so that an element in SO(4) is generated by the relation defined in Equation (9). Initial configurations for the five agents are chosen in the neighborhood of a non-consensus equilibrium to show the improvement achieved by feedback reshaping. The base case protocol with q=1 and b=1 has been simulated using the protocol derived in Equation (37) and is shown in Figure 4a. The reshaping Case 1 (q=3) protocol has also been simulated, with the appropriate $b_{n}$ coefficients. These results are shown in Figure 4b. When plotting the absolute and relative configurations between agents, the PRAs are computed as derived in Equation (8).

The results in Figure 4a show that the closed loop using the base case consensus protocol converges to the stable balanced non-consensus equilibrium with separation 2π/5 in one of the PRAs about the same plane of rotation between connected pairs, which is expected since the initial conditions are in the region of attraction for this equilibrium. There are two clusters of relative configurations, denoted by $\theta_{ij1}$, which converges towards 72°=2π/N, and $\theta_{ij2}$, which is driven to zero. This stable non-consensus equilibrium is similar to that for five agents connected by a ring graph with configurations on the unit circle, as in the Kuramoto model [17].

Figure 4b shows the principal angles associated with the relative configurations converging to zero for both PRAs as desired. The two PRAs for each agent converge to the same steady-state limits $\theta_{ss1}$ and $\theta_{ss2}$, although $\theta_{ss1}\neq \theta_{ss2}$. This is expected, since nothing is driving the agent’s configuration to an isoclinic rotation. For the five-agent ring topology and initial conditions chosen, the reshaping Case 1 protocol achieves the desired outcome. The reshaping protocol implemented in this simulation has destabilized the stable non-consensus equilibrium and resulted in the AGAS of the synchronized state.

#### 3.3.3. Nine-Agent Ring Graph

Now, we reconsider the case of N=9 agents with the undirected ring graph in Figure 1b. The initial conditions are defined in Equation (44), and the simulation is conducted using the reshaping Case 1 and Case 2 protocols.

**Figure 5:**
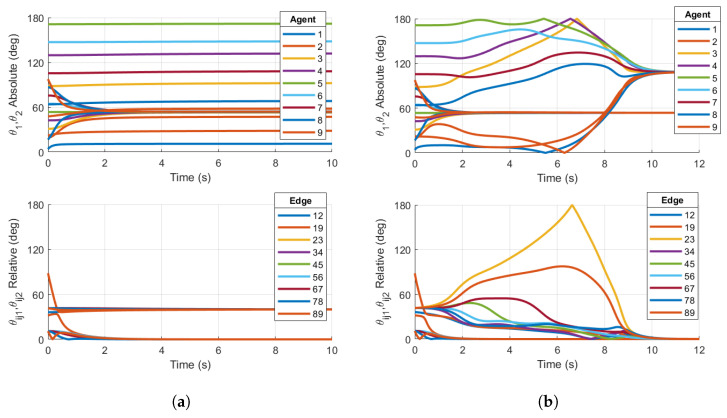
Simulations on $SO{\left(4\right)}^{9}$ for (**a**) Case 1 reshaping protocol with $\arg\max_{\theta \in [0,180]}f\left(\theta \right)=50{}^{\circ}$ on a 9-agent ring graph and (**b**) Case 2 reshaping protocol with $\arg\max_{\theta \in [0,180]}f\left(\theta \right)=35{}^{\circ}$ on a 9-agent ring graph. Initial conditions are chosen in the region of attraction of p=1 non-consensus equilibria. Right plot demonstrates consensus, while left plot does not.

Similar to the five-agent base case converging to non-consensus equilibria in Figure 4a, the nine-agent Case 1 reshaping fails to destabilize the non-consensus equilibria, resulting in a steady-state value of the relative PRA $\theta_{ij1}$ in Figure 5. This principal rotation angle $\theta_{ij1}$ converges to the separation $\theta_{ss1}=2\pi /9$ of the non-consensus balanced equilibrium. This is a result of the fact that $\theta_{ijk}<\arg\max_{\theta \in [0,180]}f_{1}\left(\theta \right)=50{}^{\circ}$. As described in Section 3.1, the Case 1 reshaping algorithm fails to destabilize the non-consensus equilibria. Since stable non-consensus equilbria remain, the resulting consensus configuration is only locally stable and not AGAS stable.

However, reshaping Case 2 successfully destabilizes the non-consensus equilibria. Figure 5b shows that all $\theta_{ijk}$ associated with the relative configurations converge to zero, signifying that consensus has been achieved. This is expected since $\theta_{ijk}=40{}^{\circ}>\arg\max_{\theta \in [0,180]}f_{2}\left(\theta \right)=35{}^{\circ}$.

The results of the nine-agent ring graph simulation confirm the conclusions in [20] for consensus on $SO{\left(3\right)}^{N}$. Case 2 feedback reshaping destabilizes non-consensus equilibria for a nine-agent ring graph and ensures the almost global stability of the consensus subspace.

#### 3.3.4. Nine-Agent Ring Lattice

Similarly to the previous sections, the undirected, nine-agent ring lattice in Figure 1c is assessed with the same initial conditions as in the nine-agent ring graph simulation. The lattice allows communication with m=2 nearest neighbors on either side of each agent.

**Figure 6:**
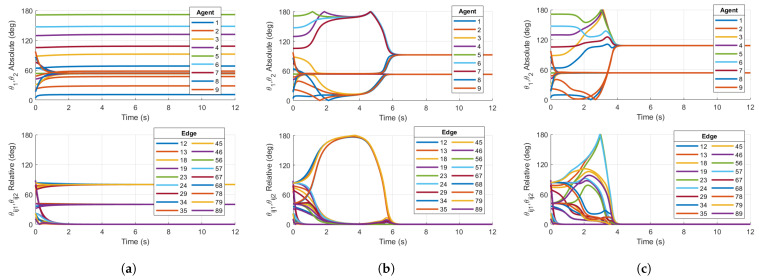
Simulations on $SO{\left(4\right)}^{9}$ for (**a**) Base case protocol on a 9-agent ring lattice, (**b**) Case 1 reshaping protocol with $\arg\max_{\theta \in [0,180]}f\left(\theta \right)=50{}^{\circ}$ on a 9-agent ring lattice, and (**c**) Case 2 reshaping protocol with $\arg\max_{\theta \in [0,180]}f\left(\theta \right)=35{}^{\circ}$ on a 9-agent ring lattice. Initial conditions are chosen in the region of attraction of p=1 non-consensus equilibria. (**b**,**c**) demonstrate consensus, while (**a**) does not.

Figure 6a shows that the base case protocol results in principal angles associated with the relative attitudes converging to separation angles 2πp/9 for p=1,2. Figure 6b shows that Case 1 reshaping successfully destabilizes the non-consensus equilibria and converges to zero relative principal rotation angles, thus showing that consensus is achieved, which is different from the result for the nine-agent ring graph. In general, the addition of connections or increasing the value of *m* for the ring lattice has the effect of decreasing the stability of the non-consensus equilibria. For a ring lattice with m>1, it has been shown [21] that there is a sequence of ring lattice networks with connectivity approaching 68.09% (i.e., the maximum node degree is 0.6809(N−1)), for which the p=1 balanced equilibrium (in which the minimum separation is 2πp/N) is still stable. Figure 6c shows that Case 2 reshaping also results in consensus, as well as a faster convergence time with respect to Case 1 in this example. This communication topology further demonstrates the reshaping method’s ability to ensure the almost global stability of the consensus subspace.

## 4. Consensus on *TSO*(4)*^N^* with Reshaping

In this section, a consensus protocol is proposed on TSO(4), the tangent bundle of SO(4). As an extension of the strategy in Section 3.1, the dynamics, which now include both kinetics and kinematics, become

(45) $$\dot{R_{i}}=R_{i}\Omega_{i}\;{\dot{\Omega }}_{i}=\tau_{i}^{\wedge }$$

where $\Omega_{i}\in \mathrm{so}\left(4\right)$ is now a state in addition to $R_{i}\in SO\left(4\right)$, and $\tau_{i}\in R^{6}$ is the control input. The second equation in Equation (45) is analogous to Euler’s equation for rigid body motion in the case of TSO(3).

### 4.1. Proposed Consensus Protocol on $TSO{\left(4\right)}^{N}$

Similar to the strategy proposed for $SO{\left(4\right)}^{N}$ in Section 3.1, the $TSO{\left(4\right)}^{N}$ reshaped Lyapunov candidate function is proposed as

(46) $$V\left(R_{j}^{T}R_{i},\Omega_{i}\right)=\frac{k_{p}}{2}\sum_{1\leq i<j\leq N}a_{ij}\sum_{n=1}^{q}\frac{b_{n}}{n}\mathrm{tr}\left(I_{4}-{\left(R_{j}^{T}R_{i}\right)}^{n}\right)+\frac{1}{4}\sum_{i=1}^{N}\mathrm{tr}\left(\Omega_{i}^{T}\Omega_{i}\right).\;$$

Using the relation ${\left(\Omega_{i}^{\vee }\right)}^{T}\Omega_{i}^{\vee }=\frac{1}{2}{∥\Omega_{i}∥}_{F}^{2}=\frac{1}{2}\mathrm{tr}\left(\Omega_{i}^{T}\Omega_{i}\right)$, the derivative of Equation (46) becomes

(47) $$\dot{V}\left(R_{j}^{T}R_{i},\Omega_{i}\right)=k_{p}\sum_{i=1}^{N}{\left(\Omega_{i}^{\vee }\right)}^{T}\sum_{j=1}^{N}a_{ij}\psi \left(R_{j}^{T}R_{i}\right)+\sum_{i=1}^{N}{\left(\Omega_{i}^{\vee }\right)}^{T}\tau_{i}.$$

Let RiΩiRiT=ΩiN represent the angular rate dyadic resolved into the inertial frame and Ω∨N=[Ω1∨TNΩ2∨TN…ΩN∨TN]T denote the stacked vector of angular rate dyadics for all agents. The relation

(48) Ω1∨N=BiΩi∨=(RiΩiRiT)∨

is used to express the Lyapunov derivative, where $B_{i}\in O\left(6\right)$ is obtained in terms of the submatrices of $R_{i}$ as

(49) $$B_{i}=\left[\begin{array}{cc} cof(R_{i11}) & {\left(R_{i12}\right)}^{\times }\\ -R_{i11}{\left(R_{i21}\right)}^{\times } & R_{i22}R_{i11}-R_{i12}R_{i21} \end{array}\right]$$

In Equation (49), cof() represents the cofactor matrix and $R_{i}$ is decomposed into submatrices as defined in Equation (31). The proposed consensus protocol in terms of the relative PRAs and angular rate dyadic resulting from Equation (52) becomes

(50) $$\tau_{i}=-k_{p}\sum_{j=i}^{N}a_{ij}\psi \left(R_{j}^{T}R_{i}\right)-k_{d}\sum_{j=i}^{N}a_{ij}\left[\Omega_{i}^{\vee }-{\left(R_{i}^{T}R_{j}\Omega_{j}R_{j}^{T}R_{i}\right)}^{\vee }\right]-k_{i}\Omega_{i}^{\vee }.\;$$

where $k_{i}>0$ for at least one value of *i* and $k_{i}=0$ otherwise. The purpose of the last term in Equation (50) is to bring the angular rate tensors to zero. Equations (45) and (50) yield the reshaped closed loop given by

(51) $${\dot{R}}_{i}=R_{i}\Omega_{i},\;{\dot{\Omega }}_{i}=-k_{p}\sum_{j=i}^{N}a_{ij}{\left[\psi \left(R_{j}^{T}R_{i}\right)\right]}^{\wedge }-k_{d}\sum_{j\neq i}a_{ij}\left[\Omega_{i}-\left(R_{i}^{T}R_{j}\Omega_{j}R_{j}^{T}R_{i}\right)\right]-k_{i}\Omega_{i}.\;$$

Furthermore, utilizing Equations (48) and (49), Equation (47) can now be expressed as

(52) V˙=−(Ω∨N)T((kdL+K)⊗I6)(Ω∨N)<0

where $K=diag(k_{1},\ldots ,k_{N})$. Since it was assumed that $k_{i}>0$ for at least one value of *i*, the matrix in Equation (52) is positive definite. The largest invariant set where the Lyapunov rate vanishes is M={(Ri,Ωi∨N):(L⊗I6)(Ω∨N)=0}. Hence, $\dot{V}=0$ when $\Omega_{i}=0$ for i=1,…,N. However, no solution can stay in the set $\left\{\left(R_{i},\Omega_{i}\right):\Omega_{i}=0_{4}\right\}$ other than when the PRAs associated with the relative configuration $R_{j}^{T}R_{i}$ are 0 or π (corresponding to consensus or partial consensus equilibria) or for the case of non-consensus equilibria, because, to obtain $\dot{\Omega }=0$, Equation (51) requires that $\sum_{j=1}^{N}a_{ij}{\left(\psi \left(R_{j}^{T}R_{i}\right)\right)}^{\wedge }=0$ for i=1,…,N. As in consensus on $SO{\left(4\right)}^{N}$, there are three possible classifications for the stability of equilibria when evaluating $\dot{V}\left(R_{j}^{T}R_{i},\Omega_{i}\right)=0$. Consensus equilibria corresponds to $R_{j}^{T}R_{i}=I_{4},\Omega_{i}=0_{4}$, implying that the relative configuration PRAs are $\theta_{ij1},\theta_{ij2}=0$ for all (i,j) pairs. Since this configuration represents the minimum in Equation (46), it is concluded that the consensus equilibrium with $\Omega_{i}=0$ is locally asymptotically stable. Partial consensus equilibria can exist here as well, as in the case of $\theta_{ijk}=\pi$ for at least one pair (i,j) for k=1 or 2 leading to an unstable set. Lastly, as in the $SO{\left(4\right)}^{N}$ case, non-consensus equilibria can exist when considering five-agent and nine-agent ring graphs, which will be demonstrated by simulation in the subsequent section. Reshaping is again used to destabilize existing non-consensus equilibria, successfully enlarging the region of attraction of the consensus manifold and possibly resulting in the AGAS stability of the consensus manifold. The local stability analysis in Section 3.2 must be used to support this claim.

### 4.2. Illustrative Examples

#### 4.2.1. Initial Conditions near Consensus

Similarly to the $SO{\left(4\right)}^{N}$ case, feedback reshaping is used to demonstrate the destabilization of non-consensus equilibria. Again, an undirected ring graph with initial conditions in the non-consensus region of attraction is assessed for the base case, reshaping Case 1, and reshaping Case 2 on $TSO{\left(4\right)}^{N}$.

**Figure 7:**
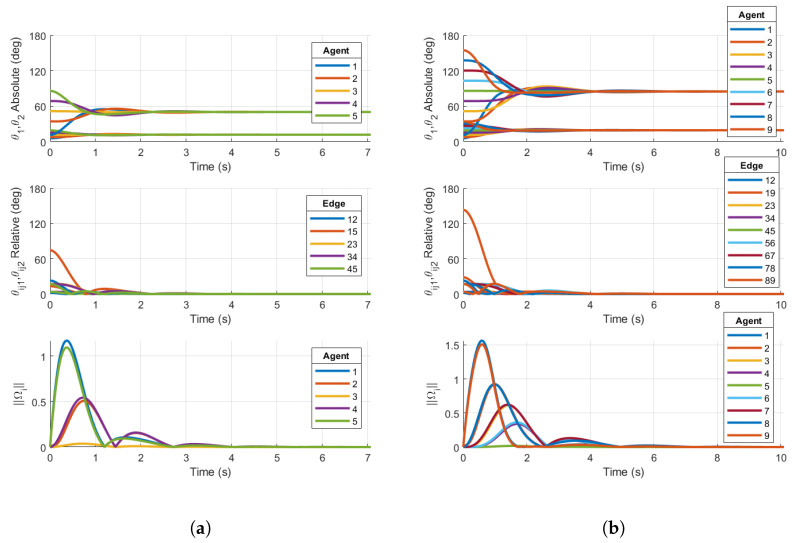
Simulations on $TSO{\left(4\right)}^{N}$ for (**a**) base case protocol on a 5-agent ring graph and (**b**) base case protocol on a 9-agent ring graph. Initial conditions are chosen within the region of attraction of the consensus manifold. Both plots demonstrate consensus.

We now show simulations on $TSO{\left(4\right)}^{N}$ that demonstrate the local stability of the consensus manifold using the base case protocol for the two ring graphs shown in Figure 1a,b. Figure 7 shows examples of the base case for both five- and nine-agent ring graphs achieving consensus. Similarly to the simulation results on $SO{\left(4\right)}^{N}$, the absolute and relative PRAs show convergence to consensus equilibria. Additionally, the norms of $\Omega_{i}$ are shown to converge towards zero. The following simulations will demonstrate that the same topologies with certain initial conditions result in convergence towards stable non-consensus equilibria.

#### 4.2.2. Five-Agent Ring Graph with Initial Conditions Away from Consensus

**Figure 8:**
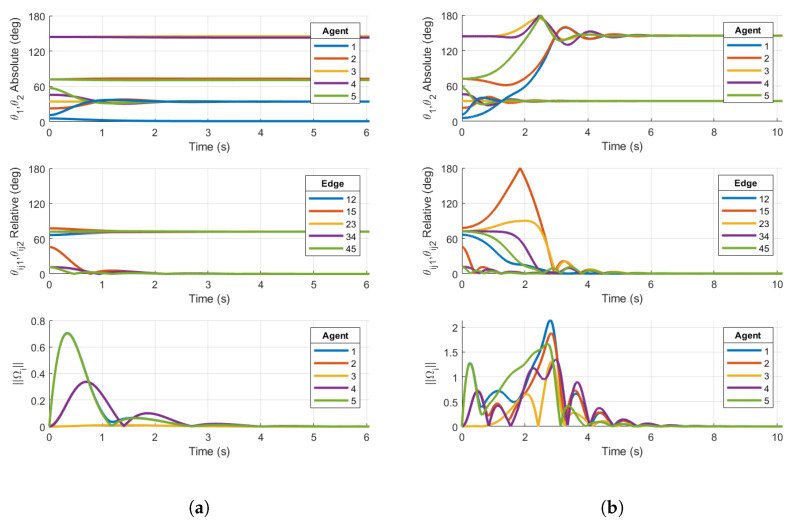
Simulations on $TSO{\left(4\right)}^{5}$ for (**a**) Base case protocol on a 5-agent ring graph and (**b**) Case 1 reshaping protocol with $\arg\max_{\theta \in [0,180]}f\left(\theta \right)=50{}^{\circ}$ on a 5-agent ring graph. Initial conditions are chosen in the region of attraction of p=1 non-consensus equilibria. Right plot demonstrates consensus, while left plot does not.

Now, we reconsider the ring graph with the N=5 agent configuration in Section 3.3.2. The base case protocol has been simulated using the consensus protocol derived in Equation (50) and is shown in Figure 8a. The reshaping Case 1 (q=3) consensus protocol has been simulated for the protocol derived in Equation (50), with the appropriate $b_{n}$ coefficients. These results are shown in Figure 8b.

The results in Figure 8a show that the closed loop using the base case consensus protocol converges to a stable, balanced non-consensus equilibrium. As in the SO(4) simulation, there are two clusters of relative configurations denoted by relative PRAs $\theta_{ij1}$, which converge towards 72°=2π/N and $\theta_{ij2}$, driven to zero. Similarly to consensus on a circle, there are known stable equilibria on a ring graph [20] with n=5 agents at 2πp/N, p=1,2 relative rotation angles on the same axis.

Figure 8b shows the relative configurations converging to zero for both $\theta_{ijk}$ as desired. The two PRAs for each agent converge to the same steady-state limits, although they do not equal each other. As in the SO(4) example, this is expected when not enforcing an isoclinic rotation. For the five-agent ring topology and initial conditions chosen, the reshaping Case 1 protocol achieves the desired outcome. The reshaping consensus protocol implemented in the simulation has destabilized the stable non-consensus equilibrium and resulted in the AGAS of the synchronized state.

#### 4.2.3. Nine-Agent Ring Graph

Now, we reconsider the case of N=9 agents with the undirected ring graph in Figure 1b. Again, the initial conditions are as defined in Equation (44), and the simulation is conducted using the reshaping Case 1 and Case 2 protocols.

**Figure 9:**
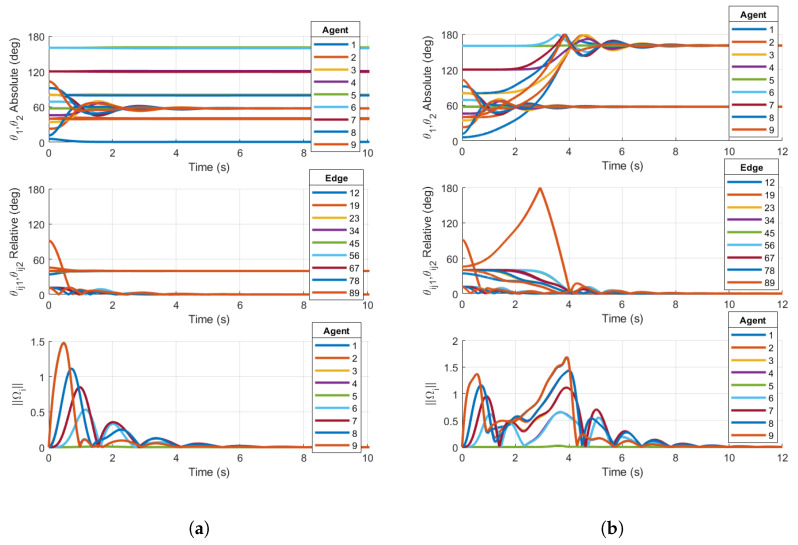
Simulations on $TSO{\left(4\right)}^{9}$ for (**a**) Case 1 reshaping protocol with $\arg\max_{\theta \in [0,180]}f\left(\theta \right)=50{}^{\circ}$ on a 9-agent ring graph and (**b**) Case 2 reshaping protocol with $\arg\max_{\theta \in [0,180]}f\left(\theta \right)=35{}^{\circ}$ on a 9-agent ring graph. Initial conditions are chosen in the region of attraction of p=1 non-consensus equilibria. Right plot demonstrates consensus, while left plot does not.

Similarly to the five-agent base case converging to non-consensus equilibria in Figure 8a, the nine-agent Case 1 reshaping fails to destabilize the non-consensus equilibria, resulting in a steady-state value of the relative PRA $\theta_{ijk}$ in Figure 9a. $\theta_{ijk}$ converges to the non-consensus equilibria 2π/N just as in the five-agent base case. This is a result of $\theta_{ijk}=40{}^{\circ}<\arg\max_{\theta \in [0,180]}f_{1}\left(\theta \right)=50{}^{\circ}$. As described in Section 3.1, the reshaping algorithm fails to destabilize the non-consensus equilibria. Since stable non-consensus equilibria remain, the resulting configuration is only locally stable and not AGAS stable.

Reshaping Case 2 successfully destabilizes the non-consensus equilibria. Figure 9b shows all $\theta_{ijk}$ converging to zero, signifying that consensus has been achieved. This is expected since $\theta_{ijk}=40{}^{\circ}>\arg\max_{\theta \in [0,180]}f_{2}\left(\theta \right)=35{}^{\circ}$. The results of the nine-agent ring graph simulation confirm the conclusions in [20]. Case 2 reshaping was designed to destabilize non-consensus equilibria and ensure the almost global stability of the consensus subspace under a communication topology of a fixed number of agents *N* on TSO(3), and this claim is justifiably extended to TSO(4).

## 5. Conclusions

Lyapunov-based consensus protocols with reshaping for multi-agent systems on $SO{\left(3\right)}^{N}$ are leveraged and extended to multi-agent configuration synchronization on $SO{\left(4\right)}^{N}$ and $TSO{\left(4\right)}^{N}$. For synchronization with non-consensus equilibria, the reshaping strategy is used to destabilize these equilibria and produce configuration synchronization with almost global asymptotic stability. The control strategy using an approach based on a Morse–Bott–Lyapunov function is supported by numerical simulations of an undirected, ring communication graph and a ring lattice. The presented simulations confirm the proposed advantages of Case 1 and Case 2 reshaping on $SO{\left(4\right)}^{N}$ and $TSO{\left(4\right)}^{N}$ synchronization by destabilizing stable non-consensus equilibria to achieve AGAS on the consensus manifolds $SO{\left(4\right)}^{N}$ and $TSO{\left(4\right)}^{N}$.

The illustrative examples used to demonstrate how reshaping destabilizes non-consensus equilibria are limited to ring graphs and ring lattices. These are only a subset of the set of circulant networks. The Kuramoto model [21] can be considered as an analog of the consensus protocols on SO(3) and SO(4) [20]. Specifically, the single-integrator Kuramoto model corresponds to kinematics-level control on SO(4) and the double-integrator Kuramoto model corresponds to TSO(4). The Kuramoto model is analyzed in [21] for circulant networks, and it is proven that there exists an upper bound on connectivity for which at least one non-consensus equilibrium is locally stable for circulant networks. Meanwhile, [30] shows that, for ring lattices, this upper bound is μ=0.6818. Thus, for a nine-node ring lattice with m=3, the consensus manifold is AGAS stable without the need for reshaping, since δ=6 and μ=0.75 in Equation (2). Similarly, for any complete graph with m=1, the consensus manifold is globally stable. As shown in Section 3.3.4, increased connectivity reduces the order of reshaping required for the nine-agent ring lattice, where Case 1, as opposed to Case 2, in the nine-agent ring graph example is sufficient in achieving consensus. Based on this observation, it can be conjectured that, if a network has a subgraph consisting of a ring graph or ring lattice on N nodes, then a reshaping strategy with $\arg\max_{\theta \in [0,180]}f\left(\theta \right)<2\pi /N$ can be developed such that almost global stability for the synchronization manifold is guaranteed.

## Data Availability

Data is contained within the article.
